# Clinical characteristics of pulmonary sarcoidosis in China: a retrospective, multicenter study

**DOI:** 10.1080/07853890.2025.2540017

**Published:** 2025-08-03

**Authors:** Kaige Wang, Linhui Yang, Zhen Kang, Zhuang Luo, Dan Liu, Fen Tan, Weimin Li

**Affiliations:** a Department of Pulmonary and Critical Care Medicine, West China Hospital, Sichuan University, Chengdu 610000, P.R. China; b Department of Radiology, Tongji Hospital, Tongji Medical College, Huazhong University of Science and Technology, Wuhan 430000, P.R. China; c Department of Pulmonary and Critical Care Medicine, The First Affiliated Hospital of Kunming Medical University, Kunming 650000, P.R. China; d Department of Critical Care Medicine, The Second Xiangya Hospital, Central South University, Changsha 410011, P.R. China

**Keywords:** Clinical features, intrathoracic lymph node tuberculosis, pulmonary sarcoidosis

## Abstract

**Background:**

Patients with pulmonary sarcoidosis or intrathoracic lymph node tuberculosis (TB) may present with comparable clinical manifestations that pose challenges in differentiation. This study aims to improve the diagnostic accuracy of pulmonary sarcoidosis.

**Methods:**

A retrospective analysis of patients diagnosed with pulmonary sarcoidosis or intrathoracic lymph node TB within the past decade at four tertiary hospitals in China was conducted. According to the inclusion and exclusion criteria, a total of 968 patients were ultimately enrolled in the study, comprising 477 individuals diagnosed with pulmonary sarcoidosis and 491 individuals diagnosed with intrathoracic lymph node TB. The analysis focused on general information, clinical manifestations, and auxiliary examination results, with a comparative analysis between the two groups.

**Results:**

The median age of onset for pulmonary sarcoidosis was 50 years, with females accounting for 68.94% of the patients. Common symptoms of pulmonary sarcoidosis included cough, sputum production, dyspnea, and chest pain, while approximately 34.12% of patients were asymptomatic. Fever, fatigue, and night sweats occurred less frequently in pulmonary sarcoidosis patients than in those with intrathoracic lymph node TB. Uveitis and myocardial sarcoidosis were observed exclusively in pulmonary sarcoidosis patients. The median time from symptom onset to the diagnosis of pulmonary sarcoidosis was up to three months. Approximately 47.29% of pulmonary sarcoidosis patients had reduced peripheral blood lymphocyte counts, and 94.12% exhibited symmetric enlargement of hilar lymph nodes on chest CT. Both pulmonary sarcoidosis and intrathoracic lymph node TB showed granulomatous inflammation, with 64.36% of intrathoracic lymph node TB cases presenting necrotic foci. Bronchoscopy was the primary method for biopsy, and only 11.06% of pulmonary sarcoidosis patients had multiple nodules in the tracheal or bronchial mucosa, with a low positivity rate for pathogen tests.

**Conclusion:**

Pulmonary sarcoidosis predominantly affects middle-aged and young women and can be differentiated from intrathoracic lymph node TB by the presence of uveitis and myocardial sarcoidosis, although these manifestations are rare. A significant proportion of pulmonary sarcoidosis patients experience a reduction in their peripheral blood lymphocyte count. Chest CT scans often reveal symmetric bilateral enlargement of hilar lymph nodes, and in some cases, multiple nodules in the tracheal or bronchial mucosa. Both pulmonary sarcoidosis and intrathoracic lymph node TB show granulomatous inflammation, but tuberculosis lesions are more likely to necrose.

## Introduction

Pulmonary sarcoidosis is a systemic granulomatous disease of unknown etiology, characterized by the presence of noncaseating epithelioid cell granulomas, which can manifest in multiple organs. The most commonly affected sites are the thoracic lymph nodes and lungs, followed by the skin and eyes [1]. The clinical presentation and prognosis of pulmonary sarcoidosis exhibit significant heterogeneity, with typical symptoms encompassing cough, expectoration, and dyspnea. However, a subset of patients may lack overt clinical manifestations; instead, imaging studies may reveal enlarged chest lymph nodes or abnormal pulmonary lesions. The majority of patients demonstrate a favorable prognosis, as evidenced by a 50% rate of spontaneous remission within three years following diagnosis. Conversely, approximately 25% of patients exhibit an unfavorable prognosis characterized by the development of chronic and progressive disease progression, culminating in irreversible lesions such as pulmonary fibrosis, cirrhosis, fatal arrhythmia, and blindness [2,3].

Notably, patients afflicted with pulmonary sarcoidosis and intrathoracic lymph node tuberculosis (TB) may both present with mediastinal lymph node enlargement, accompanied by comparable clinical manifestations that pose challenges in differentiation. Despite the increasing research on pulmonary sarcoidosis, the existing clinical studies conducted in China predominantly consist of single-center retrospective investigations with limited sample sizes [4,5]. This study aims to analyze the clinical features of pulmonary sarcoidosis and intrathoracic lymph node TB using multicenter data, providing valuable insights for the diagnosis and treatment of pulmonary sarcoidosis.

## Method

### Study design

This study retrospectively analyzed patients diagnosed with pulmonary sarcoidosis and intrathoracic lymph node TB at West China Hospital of Sichuan University, Tongji Hospital of Huazhong University of Science and Technology, First Affiliated Hospital of Kunming Medical University, and Second Xiangya Hospital of Central South University from Jan. 2012 to Jan. 2022. The study adhered to the Declaration of Helsinki and was approved by the Biomedical Review Ethics Committee of West China Hospital (Approval No.: 2022-438). And according to the Biomedical Review Ethics Committee of West China Hospital, Sichuan University, patients’ informed consent was exempt.

### Inclusion and exclusion criteria

The diagnosis of pulmonary sarcoidosis relies on the Clinical Practice Guideline issued by the American Thoracic Society in 2020 [6]. The diagnostic criteria include the following: (1) fulfillment of pertinent clinical and/or imaging features indicative of sarcoidosis; (2) confirmation of nonnecrotizing granulomatous inflammation through at least one tissue biopsy of the affected organ; and (3) exclusion of granulomatous diseases attributable to alternative etiologies. The diagnosis of intrathoracic lymph node TB is determined by the Classification of Tuberculosis and Diagnosis of Pulmonary Tuberculosis guidelines issued by the National Health and Family Planning Commission of China in 2017 [7]. The diagnostic criteria for intrathoracic lymph node TBs include: (1) the presence or absence of tuberculosis-related clinical manifestations; the detection of necrotizing granulomatous inflammation through a pathological biopsy of the thoracic lymph nodes; and (3) positive results from lymph node tissue pathology tests, such as acid-fast bacilli smears, tuberculosis cultures, or molecular nucleic acid tests for tuberculosis. The exclusion criteria were as follows: 1) patients with granulomatous diseases caused by other reasons; 2) patients with incomplete important medical record information; 3) patients with other interstitial lung diseases or pneumoconiosis other than pulmonary sarcoidosis; and 4) patients with recurrent intrathoracic lymph node TB. A total of 1249 patients diagnosed with pulmonary sarcoidosis and intrathoracic lymph node TB, including 182 patients with incomplete data (69 patients with pulmonary sarcoidosis and 113 patients with intrathoracic lymph node TB), 26 patients with recurrent intrathoracic lymph node TB, 28 patients with pneumoconiosis, 38 patients with thoracic tumors, and 7 patients with other interstitial lung diseases, were excluded from the study. Ultimately, a total of 968 patients were included in the study, comprising 477 patients with pulmonary sarcoidosis and 491 patients with intrathoracic lymph node TB.

### Clinical data collection

The study involved the collection of comprehensive patient data, including general information, clinical manifestations, and auxiliary examination results. On the basis of the observations of the trachea and bronchial lumen during bronchoscopy, the patients were categorized into the following groups: 1) those with a normal lumen and mucosa; 2) individuals exhibiting multiple nodules on the mucosa; 3) patients with lumen stenosis, foreign body attachment, or mucosal swelling and ulceration; and 4) subjects presenting with granulation-like formations resembling new organisms within the lumen.

### Statistical analysis

Since distinguishing between stage I and stage II sarcoidosis patients and intrathoracic lymph node TB is difficult, we compared the clinical, imaging, and pathologic characteristics of patients with stage I and II sarcoidosis with those of patients with intrathoracic lymph node TB. Data analysis was performed via SPSS 23 software. Continuous variables are described in terms of means and standard deviations, whereas categorical variables are described in terms of frequencies and percentages. The chi-square (χ^2^) test or Fisher’s exact probability method was used for inter group comparisons. If more than 20% of the expected frequencies are less than 5 and greater than 1 or if the minimum expected frequency is less than 1, Fisher’s exact test can be considered. In this study, we adopted a bilateral test, and *p* < 0.05 represented a statistically significant difference.

## Results

### Clinical features of patients with sarcoidosis/intrathoracic lymph node TB

**Figure 1.**
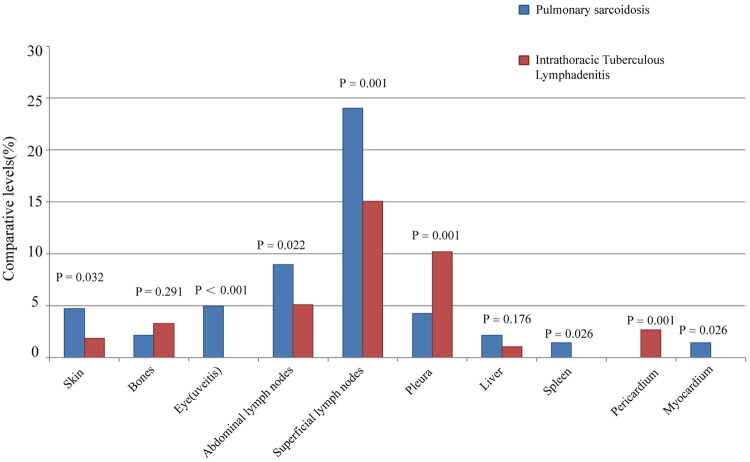
Extrapulmonary manifestations in patients with pulmonary sarcoidosis compared to intrathoracic lymph node tuberculosis

On the basis of the classification system proposed by the thoracic imaging scoring system [8], the distribution of cases in this study was as follows: 10 cases (2.1%) were categorized as stage 0, 180 cases (37.74%) as stage I, 245 cases (51.36%) as stage II, 32 cases (6.71%) as stage III, and 10 cases (2.1%) as stage IV. The median duration from symptom onset to diagnosis was 3 months for pulmonary sarcoidosis, whereas it was 2 months for intrathoracic lymph node TB. Notably, the proportion of asymptomatic cases was significantly greater in patients with pulmonary sarcoidosis than in those with intrathoracic lymph node TB. The incidence of fever, fatigue, and night sweating in patients with intrathoracic lymph node TB was significantly greater than that in patients with pulmonary sarcoidosis, as shown in Table 1. As shown in Figure 1, extrapulmonary manifestations were observed in patients. Notably, the prevalence of pulmonary sarcoidosis affecting the skin, abdominal lymph nodes, superficial lymph nodes, pleura, and spleen surpassed that of intrathoracic lymph node TB. Uveitis was observed in 21 patients (4.40%), whereas myocardial nodular disease occurred in 5 patients (1.05%). Conversely, among individuals with intrathoracic lymph node TB, only a single patient presented with eye involvement, specifically granulomatous conjunctivitis, without any manifestations in the cardiac muscle. Among patients with intrathoracic lymph node TB, 13 (2.65%) had pericardial involvement, all presenting as pericardial effusion or constrictive pericarditis, whereas patients with pulmonary sarcoidosis did not present such manifestations.

**Table 1.** Clinic characteristics of patients with pulmonary sarcoidosis or intrathoracic lymph node tuberculosis.

	Total (916)	Patients with pulmonary sarcoidosis^a^ (425)	Patients with intrathoracic lymph node tuberculosis (491)	*p* value
Age, median (IQR), years	49 (38,55)	50 (40,56)	46 (32,55)	<0.001
Sex—no.(%)				
Male	380 (41.48)	132 (31.06)	248 (50.51)	<0.001
Female	536 (58.52)	293 (68.94)	243 (49.49)	
Smoking—no. (%)	179 (19.54)	57 (13.41)	122 (24.85)	<0.001
Co-morbidities—no.(%)				
Diabetes	58 (6.33)	18 (4.24)	40 (8.15)	0.015
Hypertension	62 (6.77)	24 (5.65)	38 (7.74)	0.196
Sjogren’s syndrome	6 (0.66)	4 (0.94)	2 (0.41)	0.556
Symptom—no.(%)				
Fever	133 (14.52)	18 (4.24)	115 (23.42)	<0.001
Cough	515 (56.22)	228 (53.65)	287 (58.45)	0.144
Sputum	235 (25.67)	102 (24)	133 (27.09)	0.286
Fatigue	80 (8.73)	26 (6.12)	54 (11)	0.009
Night sweat	79 (8.62)	17 (4)	62 (12.63)	<0.001
Chest tightness	36 (3.93)	15 (3.53)	21 (4.28)	0.561
Chest pain	142 (15.5)	60 (14.12)	82 (16.7)	0.016
Dyspnea	167 (18.23)	89 (20.94)	78 (15.89)	0.048
Hemoptysis	30 (3.28)	10 (2.35)	20 (4.07)	0.145
Asymptomatic	245 (26.75)	145 (34.12)	100 (20.37)	<0.001
Time from onset to diagnosis, median (IQR), months	2.5 (1,6)	3 (1,10)	2 (1,5)	<0.001

y: year, IQR: interquartile range; *p* < 0.05 represents a statistically significant difference.

^a^ It specifically refers to patients with stage I and stage II pulmonary sarcoidosis.

### Peripheral blood characteristics of patients with sarcoidosis/intrathoracic lymph node TB

The results of peripheral blood cell and immune examinations for patients diagnosed with pulmonary sarcoidosis and intrathoracic lymph node TB are presented in Table 2. Among the patients with pulmonary sarcoidosis in stage I and II, 201 (47.29%) exhibited a decreasing lymphocyte count, whereas 153 (31.16%) patients with intrathoracic lymph node tuberculosis presented the same trend (*p* < 0.05). Specifically, 86 (62.32%) pulmonary sarcoidosis patients and 51 (42.86%) intrathoracic lymph node TB patients had reduced CD4+ T lymphocytes (*p =* 0.47). Additionally, 78 patients (56.52%) with pulmonary sarcoidosis and 76 patients (63.87%) with intrathoracic lymph node TB demonstrated a decrease in the CD8+ T lymphocyte count (*p* < 0.05). The immunoglobulin levels in the peripheral blood of the patients are presented in Table 2. Among the patients, 24 (5.65%) were found to have positive serum immune tests for pulmonary sarcoidosis, whereas 12 (2.44%) tested positive for intrathoracic lymph node TB (*p* < 0.05). Additionally, 39 (9.18%) patients with pulmonary sarcoidosis and 384 (78.21%) patients with intrathoracic lymph node TB tested positive for tuberculosis immunology (PPD skin test or TB-IGRA), resulting in a statistically significant difference (*p* < 0.001).

**Table 2.** Analysis of the patient’s peripheral blood lymphocytes and autoimmune tests.

Peripheral blood test	Total	Patients with pulmonary sarcoidosis^a^	Patients with intrathoracic lymph node tuberculosis	*p* value
Peripheral blood lymphocytes, *n*	916	425	491	
<1.1 × 10^9^/L	354	201(47.29)	153(31.16)	<0.001
T-cell subset, *n*	257	138	119	
CD4 + T lymphocytes, no. (%)				
>404/µl	120 (46.69)	52 (37.68)	68 (57.14)	0.47
≤404/µL	139 (54.09)	86 (62.32)	51 (42.86)	
CD8 + T lymphocytes, no. (%)				
>220/µL	103 (40.1)	60 (43.48)	43 (36.13)	0.01
≤220/µL	154 (59.9)	78 (56.52)	76 (63.87)	
Serum immunoglobulin, *n*	355	165	190	
IgA—no. (%)				
<836 g/L	7 (1.97)	4 (2.42)	3 (1.58)	0.854*
836–2900 g/L	221 (62.25)	103 (62.42)	118 (62.11)	
>2900 g/L	127 (35.77)	58 (35.15)	69 (36.32)	
IgG—no. (%)				
<8 g/L	8 (2.25)	4 (2.42)	4 (2.11)	0.002*
8–15.5 g/L	249 (70.14)	130 (78.79)	119 (62.63)	
>15.5 g/L	98 (27.6)	31 (18.79)	67 (35.26)	
IgM—no. (%)				
<700 g/L	45 (12.68)	19 (11.52)	26 (13.68)	0.855
700–2200 g/L	272 (76.62)	128 (77.58)	144 (75.79)	
>2200 g/L	38 (10.7)	18 (10.91)	20 (10.53)	
IgE—no. (%)				
<5 g/L	16 (4.51)	12 (7.27)	4 (2.14)	0.008
5–150 g/L	260 (73.24)	125 (75.76)	135 (70.59)	
>150 g/L	79 (22.25)	28 (16.97)	51 (27.27)	
Serum immune tests, n	916	425	491	
Positive—no. (%)	36 (3.93)	24 (5.65)	12 (2.44)	<0.001
ANA 1:100—no.(%)	32 (3.5)	22 (5.18)	10 (2.04)	NA
ANA ≥1:320—no.(%)	17 (1.86)	10 (2.35)	7 (1.42)	NA
sDNA—no.(%)	1 (0.11)	1 (0.24)	0	NA
Anti-ENA antibody				
RO-52—no. (%)	6 (0.62)	5 (1.05)	1 (0.2)	NA
Jo-1—no. (%)	1 (0.1)	1 (0.21)	0	NA
SCL-70—no.(%)	1 (0.1)	1 (0.21)	0	NA
SSA (SSB)—no.(%)	15 (1.55)	9 (1.89)	6 (1.22)	NA

ANA: antinuclear antibody, sDNA: double-stranded deoxyribonucleic acid, SCL-70: scleroderma 70.

* Fisher’s exact test; *p* < 0.05 represents a statistically significant difference in this study.

^a^ It specifically refers to patients with stage I and stage II pulmonary sarcoidosis.

### Imaging characteristics of patients with sarcoidosis/intrathoracic lymph node TB

**Figure 2.**
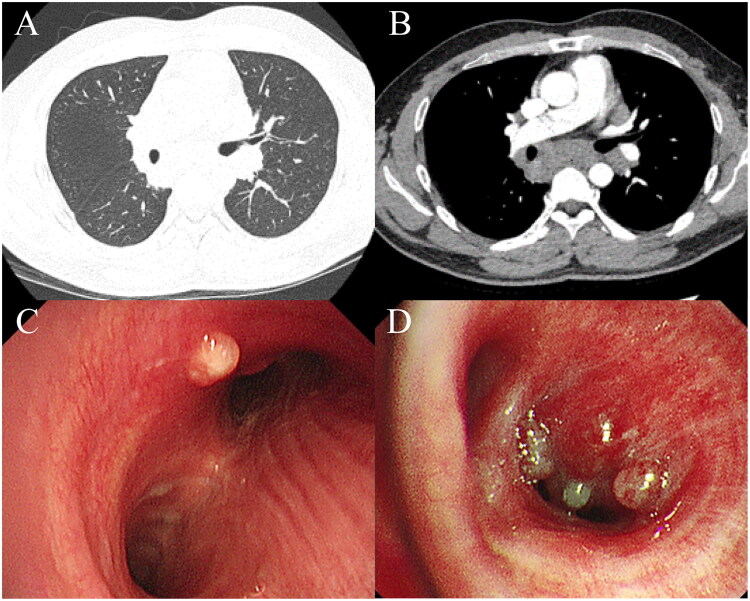
Imaging and bronchoscopic manifestations in pulmonary sarcoidosis. (A, B) Contrast-enhanced CT scans showing predominantly homogeneous enhancement, with only 4% of cases exhibiting ring-like enhancement. (C, D) Bronchoscopic views illustrating multiple nodules on the tracheal or bronchial mucosa observed in pulmonary sarcoidosis patients.

The imaging findings of the patients are presented in Table 3, indicating that 246 (57.88%) individuals with pulmonary sarcoidosis exhibited lung involvement, which aligns with the characteristics of intrathoracic lymph node TB. A total of 94.12% of patients with pulmonary sarcoidosis presented with symmetrical enlargement of hilar lymph nodes. On contrast-enhanced CT scans, only 4% exhibited ring-like enhancement, while most displayed homogeneous enhancement (Table 3; Figures 2A and 2B). Bronchoscopy remains the primary diagnostic biopsy technique for both conditions, with 408 cases (85.53%) of pulmonary sarcoidosis and 465 cases (94.7%) of intrathoracic lymph node TB being diagnosed through this procedure. Shallow lymph node biopsy, percutaneous lung puncture, and VATS serve as important supplementary diagnostic methods for pulmonary sarcoidosis, with 18 patients (3.78%), 13 patients (2.73%), and 39 patients (8.18%) respectively. Similarly, for intrathoracic lymph node TB, there were 7 patients (1.43%), 11 patients (2.24%), and 12 patients (2.44%) respectively. Among patients with pulmonary sarcoidosis, 370 individuals (87.06%) presented no abnormalities in the trachea or bronchial lumens, whereas 391 patients (79.63%) presented intrathoracic lymph node TB. Notably, 47 (11.06%) pulmonary sarcoidosis patients presented with multiple nodules in the tracheal or bronchial mucosa, as illustrated in Figure 2C and D.

**Table 3.** Imaging and bronchoscopic presentation of the patients.

	Total (916) no. (%)	Patients with pulmonary sarcoidosis^a^ (425), no.(%)	Patients with intrathoracic lymph node tuberculosis (491), no.(%)	*p* value
Manifestations of chest CT				
Infiltrative lesions in the lungs	574(59.3)	246 (57.88)	287 (58.45)	0.587
Symmetrical enlargement of hilar lymph nodes	557(57.54)	400 (94.12)	142 (28.92)	<0.001
Low-density necrosis in the center of enlarged lymph nodes	150(15.5)	11 (2.59)	139 (28.31)	<0.001
Ring enhancement in enhanced scanning	191(19.73)	17 (4)	174 (35.44)	<0.001
Calcification of lymph nodes	112(11.57)	15 (3.53)	97 (19.76)	<0.001
Bronchoscopic presentation				
Normal	787 (85.92)	370 (87.06)	391 (79.63)	0.177
Multiple nodules on membranes	58 (6.33)	47 (11.06)	9 (1.83)	<0.001
Narrowed lumen, swollen or ulcerated mucosa	99 (10.81)	24 (5.65)	75 (15.27)	<0.001
Scar-like changes in lumen	4 (0.44)	0	4 (0.81)	0.048
Necrotic material adherence	16 (1.75)	1 (0.24)	15 (3.05)	0.001
Neoplastic organisms in the lumen	21 (2.29)	5 (1.18)	16 (3.26)	0.018

^a^ It specifically refers to patients with stage I and stage II pulmonary sarcoidosis.

*p* < 0.05 represents a statistically significant difference.

### Pathological characteristics of patients with sarcoidosis/intrathoracic lymph node TB

Pulmonary sarcoidosis exhibited pathological alterations characterized by granulomatous inflammation (93.08%) and granulomatous inflammation accompanied by noncaseous necrosis (6.92%). Among the patients diagnosed with intrathoracic lymph node TB, 251 (51.12%) presented with granulomatous inflammation with extensive lesions. Acid resistance, TB-DNA, and tuberculosis culture tests yielded positive results in 4, 5, and 0 patients with pulmonary sarcoidosis, respectively. Tuberculosis-associated examinations were conducted on the pathological tissues of individuals diagnosed with intrathoracic lymph node TB, revealing that 91 patients (18.53%) presented positive acid-fast staining, 183 patients (37.27%) presented positive TB-DNA, and 36 patients (7.33%) presented positive tuberculosis cultures. No instances of positive findings were observed for pathogens other than *Mycobacterium tuberculosis*.

## Discussion

In clinical practice, pulmonary sarcoidosis may manifest with typical symptoms associated with respiratory system diseases, including cough, expectoration, chest pain, and dyspnea. Importantly, however, some patients may be asymptomatic. To establish a diagnosis, it is necessary to exclude other granulomatous diseases on the basis of pathological confirmation of noncaseating necrotizing granulomatous inflammation [9]. Both pulmonary sarcoidosis and intrathoracic lymph node TB are frequently mediastinal diseases that exhibit nonspecific clinical manifestations, and are often characterized by enlarged thoracic lymph nodes and, in some cases, lung infiltration [10–12]. This study included 477 patients with pulmonary sarcoidosis admitted to 4 medical centers in different regions of China in the past 10 years, and 491 patients with intrathoracic lymph node TB admitted during the same period as controls to analyze their clinical characteristics.

This study revealed that the median age of onset in individuals diagnosed with pulmonary sarcoidosis is 50 years, with females comprising 68.97% of the patient population. These findings align with previous reports in the European and American literature [13]. Moreover, the incidence of fever, fatigue, and night sweating was notably greater in patients with intrathoracic lymph node TB than in those with pulmonary sarcoidosis. Additionally, the proportion of asymptomatic individuals diagnosed with pulmonary sarcoidosis was significantly greater than that observed in patients with intrathoracic lymph node TB. The prevalence of cutaneous, abdominal lymph node, superficial lymph node, pleural, and splenic involvement is greater in patients with pulmonary sarcoidosis than in those with intrathoracic lymph node TB. Furthermore, patients with pulmonary sarcoidosis may exhibit uveitis and myocardial involvement, whereas this manifestation may be absent in patients with intrathoracic lymph node TB. Conversely, pericardial involvement (pericardial effusion or constrictive pericarditis) may occur in patients with intrathoracic lymph node TB, but is not observed in patients with pulmonary sarcoidosis. It is postulated that uveitis and myocardial involvement exhibit greater specificity as extrapulmonary manifestations of sarcoidosis, whereas pericardial involvement demonstrates greater specificity as a manifestation of intrathoracic lymph node TB, thereby aiding in the differentiation between these two diseases.

Previous studies have demonstrated a reduction in the peripheral blood lymphocyte count in approximately 50% of sarcoidosis patients, indicating a correlation with the progression of chronic disease [14]. Extensive research has established that pulmonary sarcoidosis is a systemic ailment triggered by various factors that disrupt immune function, ultimately resulting in diminished or exhausted T cell activity [15,16]. Scholarly investigations have further revealed a decrease in peripheral blood CD4+ T and CD8+ T lymphocytes in individuals afflicted with pulmonary sarcoidosis, which is directly associated with the severity of sarcoidosis and the extent of pulmonary function decline. Some scholars have suggested that the decline in CD4+ T cells can be attributed to the migration of CD4+ T cells from the bloodstream back to the mediastinal lymph nodes [17,18]. In the present study, a notable proportion of patients with pulmonary sarcoidosis (47.17%) exhibited a reduction in the overall lymphocytes count in the peripheral blood, with 57.14% and 50.65% of patients experiencing decreases in peripheral blood CD4+ T lymphocytes and CD8+ T lymphocytes, respectively. Similarly, patients with intrathoracic lymph node TB demonstrated a decline in the total counts of peripheral blood lymphocytes, CD4+ T lymphocytes and CD8+ T lymphocytes, with percentages of 31.16%, 42.86% and 63.87%, respectively. Research findings indicate that tuberculosis infection can lead to a reduction in the consumption of peripheral blood lymphocytes [19]. The underlying cause and significance of heightened blood immunoglobulin levels in individuals with pulmonary sarcoidosis remain uncertain [20]. Within the scope of this study, 5.66% of patients diagnosed with pulmonary sarcoidosis presented positive results in serum autoimmune tests. The prevalent antibodies detected were anti SSA antibodies, anti-nuclear antibodies, and anti-RO-52 antibodies, aligning with previously documented cases [4,21]. Therefore, the immunological mechanisms involved in the occurrence and development of sarcoidosis need further in-depth research.

Numerous studies have been conducted on the imaging manifestations of individuals diagnosed with pulmonary sarcoidosis. Yang Zhigang et al. [22] demonstrated that enhanced CT scans frequently reveal uniform enhancement of enlarged thoracic lymph nodes in these patients, which tend to be symmetrically distributed on both lung hili. The present study revealed a greater prevalence of hilar lymph node enlargement in patients with pulmonary sarcoidosis than in those with intrathoracic lymph node tuberculosis, with a significantly greater likelihood of symmetrical hilar lymph node enlargement (87% vs 18.92%). The findings of this study indicate that intrathoracic lymph node TB is more susceptible to low-density necrosis, circular enhancement, and lymph node calcification located centrally within the lymph node. This research highlights bronchoscopy as the primary diagnostic approach for pulmonary sarcoidosis and intrathoracic lymph node TB. Patients with pulmonary sarcoidosis commonly exhibit multiple nodules in the trachea or bronchial mucosa. The presence of bronchial stenosis, mucosal swelling or ulceration, scar-like changes in the lumen, necrotic adherings, or new biological granulation within the bronchial lumen often suggests the co-occurrence of intrathoracic lymph node TB and tracheal or bronchial tuberculosis.

In addition to histopathological examination, pathogen detection serves as a crucial approach for distinguishing between pulmonary sarcoidosis and intrathoracic lymph node TB. Within the scope of this study, the rate of positive pathogen tests among patients with intrathoracic lymph node TB was only 37.27%. Sahajal et al [23]. conducted an analysis on tissue samples obtained from 147 individuals who were diagnosed with pulmonary sarcoidosis and intrathoracic lymph node TB; there samples specifically targeted *Mycobacterium tuberculosis* and rifampicin resistance genes. The findings indicated that the diagnosis of intrathoracic lymph node TB exhibited a high level of specificity (97.9%) and positive predictive value (92.9%), albeit with a sensitivity of only 49.1%. Furthermore, 4.61% of the individuals diagnosed with pulmonary sarcoidosis in this investigation presented a documented history of tuberculosis. Additionally, 9.01% of the patients had positive results in tuberculosis immunological tests, indicating either past tuberculosis infection or latent tuberculosis infection. These findings align with those of previous studies [24,25]. Notably, certain investigations have demonstrated that the likelihood of tuberculosis progressing to sarcoidosis is 8.09 times greater than that of the general population, thus implying that tuberculosis infection is a significant risk factor for the development of sarcoidosis [26].

## Conclusion

Pulmonary sarcoidosis is frequently observed in young and middle-aged females, wherein uveitis and myocardial sarcoidosis serve as distinctive manifestations that differentiate it from intrathoracic lymph node TB. A considerable proportion of individuals with pulmonary sarcoidosis exhibit a reduction in peripheral blood lymphocyte count, whereas approximately half experience a decrease in peripheral blood CD4+ T cells and CD8+ T cells. Additionally, certain patients are positive for autoimmune antibodies, suggesting the involvement of autoimmune reactions in the pathogenesis of the disease. Chest CT commonly reveals bilateral symmetric hilar lymph node enlargement, accompanied by uniform enhancement on enhanced scans. Certain patients may exhibit multiple nodules in the tracheal or bronchial mucosa. Both pulmonary sarcoidosis and intrathoracic lymph node TB can display granulomatous inflammation. The rate of positive findings in pathogenic examination is low, and tuberculosis foci are more prone to exhibit large necrotic foci. Distinguishing between these conditions can be challenging.

## Data Availability

The datasets used and/or analyzed in the present study are available from the corresponding author upon reasonable request.
